# Recent and Projected Trends in Oral Tongue Cancer in the United States: A Demographic Shift in Case Burden as Early Onset Increases Among Females Subside

**DOI:** 10.21203/rs.3.rs-3359293/v1

**Published:** 2023-09-22

**Authors:** Todd Burus, Haluk Damgacioglu, Bin Huang, W. Jay Christian, Pamela C. Hull, Amanda R. Ellis, Susanne M. Arnold, Ashish A. Deshmukh, Krystle A. Lang Kuhs

**Affiliations:** Markey Cancer Center; Medical University of South Carolina; Markey Cancer Center; University of Kentucky; Markey Cancer Center; University of Kentucky; Markey Cancer Center; Medical University of South Carolina; Markey Cancer Center

**Keywords:** Oral tongue cancer, incidence, birth cohort

## Abstract

**Background:**

Oral tongue cancer (OTC) incidence has increased rapidly among young (< 50 years) non-Hispanic White (NHW) individuals in the United States (U.S.) over the last two decades; however, it is unknown if age-associated trajectories have persisted. Furthermore, incidence trends for all 50 U.S. states and the District of Columbia have never been investigated.

**Materials and methods:**

Using U.S. Cancer Statistics data, we investigated incidence trends from 2001–2019, overall and according to age, sex, race/ethnicity, and state of residence. We used age-period-cohort analysis to explore temporal patterns among birth cohorts and to project future trends and case counts.

**Results:**

OTC incidence increased across all age, sex, and racial/ethnic groups, with marked increases observed among the NHWs (2.9%/year; 95%CI, 2.2%-3.7%). Incidence among NHWs increased in most U.S. states, particularly in the Southeast. Increases were significantly greater among NHW females compared to males (3.6%/year vs 2.6%/year; *P* = 0.022). Increases among females aged 50–59 years were most notable and significantly outpaced increases among younger females (4.8%/year [95% CI, 4.1%-5.4%] vs. 3.3%/year [95% CI, 2.7%-3.8%]; *P* < .001). While both NHW male and female birth cohorts from 1925 to 1980 saw sustained increases, rates stabilized among females born after 1980. Should trends continue, the burden of new OTC cases among NHWs in the U.S. is projected to shift to older individuals (33.1% versus 49.3% aged ≥ 70) and females (86% case increase versus 62% among males).

**Conclusion:**

The period of rapidly increasing OTC incidence among younger NHW females in the U.S. is tempering and giving way to greater increases among older females, suggesting that a birth cohort effect may have influenced previously observed trends. Recent increases among NHWs aged ≥ 50 of both sexes have matched or outpaced younger age groups. Continuing increases among older individuals, particularly females, will lead to a shift in the OTC patient profile over time.

## INTRODUCTION

Over the past 25 years, head and neck cancer incidence has been declining in the United States (U.S.) with decreasing prevalence of tobacco use.^1^ Two head and neck cancer subsites—oropharyngeal cancer squamous cell carcinoma (OPSCC) and oral tongue cancer (OTC)—have been the exception. The incidences of OPSCC and OTC have increased markedly in the U.S. for several decades.^2–4^ The rise in OPSCC incidence has been attributed to human papillomavirus infection (HPV).^2^ A corresponding cause for OTC has yet to be identified.^4,5^

The rising incidence of OTC in the U.S. was first recognized to be occurring among young non-smokers.^6^ Subsequent studies over the past two decades confirmed a rapid rise in OTC incidence among young non-Hispanic White (NHW) individuals, particularly among young females without the traditional risk factors of heavy tobacco or alcohol use.^3,7^ Similar findings have been reported globally.^8–12^ This has led many in the field to consider early onset OTC (diagnosed at < 50 years) as a distinct clinical entity, with research primarily focused on understanding the differences between early versus typical onset (diagnosed at ≥ 50 years) OTC.^3,4,8,12^ Previous U.S. studies quantifying OTC incidence trends in the U.S. used the National Cancer Institute’s (NCI) Surveillance, Epidemiology, and End Results (SEER) Program data, which captures at most 48% of the U.S. population.^3,4,8,13,14^ The relative rarity of OTC combined with the limited size of the SEER Program database has restricted the ability to study more granular patterns by age and geographic region, potentially masking important information about the geographic distribution of cases and evolving age trends. With respect to OPSCC, recent studies have reported that the increasing incidence among younger individuals has moderated and the case burden is now shifting to older age groups.^15^ It remains unknown if OTC is also following a similar pattern.

The objective of this study was to provide a comprehensive analysis of recent temporal and geographic trends in OTC incidence in the U.S. and to project future case burden. To accomplish this, we used the 2001–2019 U.S. Cancer Statistics (USCS) Public Use Database, which covers 98% of the U.S. population, allowing us to consider trends in narrower age groups across both sexes and provide the first state-level analysis of OTC incidence.

## MATERIALS AND METHODS

### Data Sources

We analyzed incident cancer cases from the USCS Public Use Database for the years 2001 through 2019.^16^ This database contains submissions from the Center for Disease Control and Prevention’s (CDC) National Program of Cancer Registries (NPCR) and the NCI SEER program, covering approximately 98% of cancer cases in all 50 states and the District of Columbia during this time.

### Study Population

We identified oral tongue cancer cases using topography codes C02.0–02.3 from the International Classification of Diseases for Oncology version-3 (ICD-O-3).^17^ We excluded cases classified as C02.8 (Overlapping lesion of tongue) and C02.9 (Malignant neoplasm: Tongue, unspecified) to avoid possible misclassification of oropharyngeal base of tongue tumors as oral tongue tumors. Staging was applied according to SEER Summary Stage 2000. We included only malignant tumors and of all histological subtypes. This study did not use personal identifying information from the USCS Public Use Database or involve interaction with human subjects; and thus, was deemed exempt from institutional review by the University of Kentucky Institutional Review Board.

We analyzed patient demographics using the following variables: age at time of diagnosis, sex, race/ethnicity, and state of residence at time of diagnosis. Due to limited case numbers, available racial/ethnic categories were reduced to Hispanic, non-Hispanic Black (NHB), non-Hispanic White (NHW), and other non-Hispanic races (Other NH). For some analyses we further categorized state of residence into Southeast, Southwest, Northeast, Midwest, Rocky Mountain, Pacific, and Non-contiguous regions.

### Statistical Analysis

Incidence rates were age-adjusted to the 2000 U.S. standard population and given per 100,000 persons using SEER*Stat version 8.4.0. Rates were suppressed for any subpopulation and year/year range containing fewer than 16 cases. We estimated OTC incidence rates by sex, age, race/ethnicity, stage at diagnosis, and region of residence at diagnosis. We used Joinpoint software version 4.9.1.0 to calculate trends in OTC annual incidence rates and to estimate annual percentage changes (APC) and average APCs (AAPC).^18^ Joinpoint regression fits piecewise log-linear trends to the rates using a permutation test and provided estimates for APC of segments and AAPC as a weighted average of the APCs. To determine whether the AAPC trends were different from zero, a t test was conducted for zero joinpoints, and a z test was conducted for one or more joinpoints. Statistical significance was assessed at a level of *P* < 0.05, and all hypotheses were two-sided. For the state-level analysis, we estimated OTC incidence rates for 2001–2005 and 2015–2019 by sex, and state of residence at diagnosis. Temporal changes were quantified using incidence rate ratios (IRR).

We performed age-period-cohort analysis to estimate OTC trends by age, period, and birth cohort. We accessed case counts and population estimates for the 5-year ranges of 2005–2009, 2010–2014, and 2015–2019 from the USCS Public Use Database in SEER*Stat. The use of 13 age groupings and three calendar periods resulted in 15 partially overlapping birth cohorts (age-period-cohort analysis was restricted to ages 20 and older to ensure non-zero case counts in all age groups used). Age-period-cohort models were built using the Age Period Cohort Analysis web tool created by the NCI.^19^ To project the future burden of OTC, we used the same case counts and population estimates as above along with population projections for the 5-year ranges of 2020–2024, 2025–2029, and 2030–2034 from the U.S. Census Bureau via CDC WONDER.^20^ Case projection estimates were calculated using the *n* or *dpred* package (version 1.1) with Poisson link function in the R statistical programming language (version 4.0.5).^21,22^ Sensitivity analysis was performed on case projections by using the same model on data from 2001–2015 to project the average annual number of new cases for 2016–2020. This was compared to the average annual number of new cases observed in 2016–2019 (Additional File 1: Figure S1). For males, the *n* or *dpred* model fit to the 2001–2015 data predicted the average annual observed case count to within 4% for all but one age group, and all age groups within 8%. For females, the *n* or *dpred* model predicted the average annual observed case count to within 5% for all but one age group, and all age groups within 10%. The ‘Under 30 years’ age group was omitted from sensitivity analysis because case counts used for projections were known underestimates due to suppression.

## RESULTS

### Patient Characteristics

There were 58,661 new cases of OTC identified between the years of 2001 and 2019. Men (57.6%), non-Hispanic Whites (83.7%), those aged 60 years or older (58.0%), and individuals with localized stage disease at diagnosis (62.7%) comprised the majority of cases (Additional File 2: Table S1).

### OTC Incidence by Sex, Age, and Race/Ethnicity

**Figure 1:**
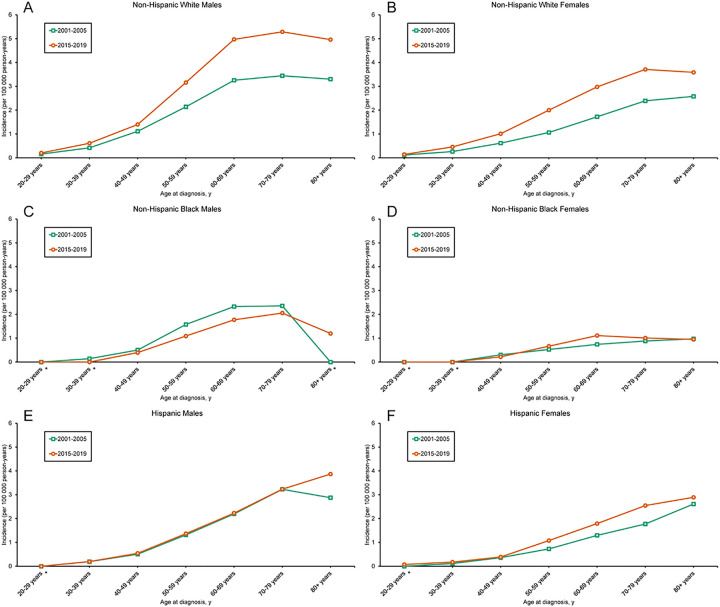
Age- and race-specific incidence rates of oral tongue cancer. Age-specific OTC incidence rates according to age at diagnosis during the calendar periods 2001–2005 and 2015–2019. Incidence rates among (A) non-Hispanic White males, (B) non-Hispanic White females, (C) non-Hispanic Black males, (D) non-Hispanic Black females, (E) Hispanic males, and (F) Hispanic females. (*) Data suppressed because there were fewer than 16 cases in the time interval.

While rates across all combinations of sex and race/ethnicity generally increased with age, the most notable increases were observed among NHWs (Fig. 1). Between 2001 and 2019, OTC incidence among NHW persons increased 2.9% per year (95% CI, 2.2–3.7%; Table 1). OTC incidence rate ratios increased among all age groups 30 years and older for both NHW males and females between 2001–2005 and 2015–2019 (Additional File 3: Table S2). OTC incidence remained largely the same among other racial/ethnic groups over the same period. Given the presence of substantial OTC incidence rate increases across NHW sexes and age groups compared to other races/ethnicities, all subsequent analyses were restricted to NHWs.

### State-specific OTC Incidence among NHWs

**Figure 2:**
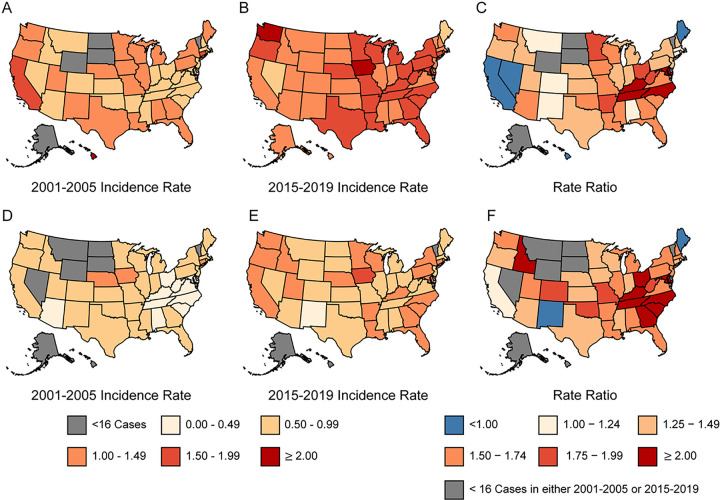
State-specific incidence rates and rate ratios for OTC among NHW males and females. (A) male incidence rate 2001–2005, (B) male incidence rate 2015–2019, (C) male rate ratios, (D) female incidence rate 2001–2005, (E) female incidence rate 2015–2019, (F) female rate ratios. Cases diagnosed in Mississippi in 2001–2002 and in Nevada in 2018– 2019 are not available. NHW: Non-Hispanic White; OTC: Oral tongue cancer.

State-specific OTC incidence increased in nearly all states for both NHW males and females, with the five highest relative increases among both sexes occurring in states from the Southeast region (Fig. 2; Additional File 4: Table S3, Additional File 5: Table S4 and Additional File 6: Figure S2). For males, the five states with the highest relative increases were Maryland (IRR = 2.4), Tennessee (IRR = 2.2), Kentucky (IRR = 2.2), North Carolina (IRR = 2.1), Arkansas (IRR = 2.0). The top five states for females were Kentucky (IRR = 3.0), South Carolina (IRR = 2.8), Maryland (IRR = 2.8), North Carolina (IRR = 2.6), and Tennessee (IRR = 2.5). Fifteen additional states had IRRs for males in excess of 1.5, as did 21 additional states for females. Relative declines in OTC incidence were observed in only four states (California, Maine, Nevada, Hawaii) for males and two states (New Mexico and Maine) for females. These findings are consistent with APC estimates on regions with available data (Additional File 7: Table S5). The most marked increases occurred in the Southeast where OTC incidence rose by 4.3% per year among males and by 5.5% per year among females.

### Stage-specific Trends in OTC Incidence among NHWs

Significant increases in local and regional stage disease were observed among both NHW males and females (Additional File 8: Figure S3). Notably, regional stage disease increased 5.1% per year (95% CI, 3.7–6.6%) among females and 4.0% (95% CI, 2.8–5.2%) among males.

### Sex- and Age-specific Trends in OTC Incidence among NHWs

**Figure 3:**
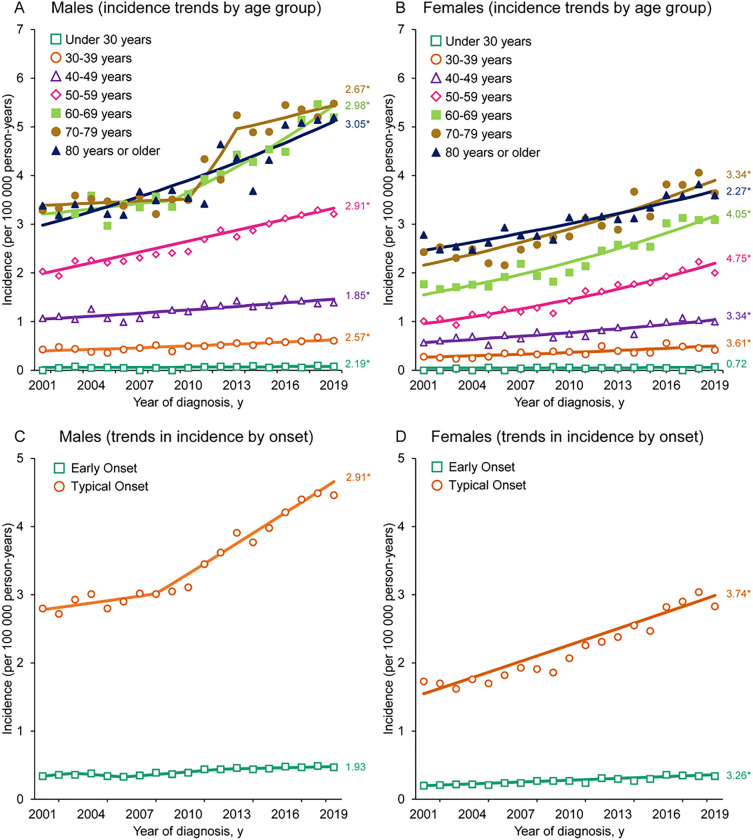
Trends in the annual incidence rates of OTC among NHW males and females. Incidence rates between 2001 and 2019 among (A) males by age groups, (B) females by age groups, (C) males by onset, and (D) females by onset. (*) indicates statistically Significant average annual percentage change in incidence for 2001–2019. NHW: Non-Hispanic White; OTC: Oral tongue cancer.

Significantly greater increases occurred among females (AAPC 3.6%; 95% CI, 3.2–4.0%) than among males (AAPC 2.6%; 95% CI, 1.8–3.4%; pairwise comparison *P* = 0.022) (Table 1). Stratifying by age, NHW males across all 10-year age groups experienced Significant increases in OTC incidence, while incidence increased in all NHW female age groups except those under 30 years (*P* = 0.565) (Figs. 3A and 3B; Additional File 9: Table S6). The most notable age-specific incidence rate (ASIR) increase occurred among females aged 50–59 years (AAPC 4.8%; 95% CI, 4.1–5.4%).

Early onset OTC incidence (< 50 years) only increased significantly among females (AAPC 3.3%; 95% CI, 2.7–3.8%) while typical onset incidence (≥ 50 years) saw Significant increases for both males (AAPC 2.9%; 95% CI, 2.2–3.6%) and females (AAPC 3.7%; 95% CI, 3.3–4.2%) (Fig. 3C and 3D; Additional File 9: Table S6). In particular, incidence rate increases among NHW females aged 50–59 years significantly outpaced increases among NHW females aged < 50 years (AAPC 3.3%; 95% CI, 2.7–3.8%; pairwise comparison *P* < 0.001), resulting in a notable shift in case burden (Additional File 10: Figure S4). There was no corresponding Significant difference between early onset male incidence trends and the increases seen among males aged 50–59 years (*P* = 0.444).

### Cohort-specific Trends in OTC Incidence among NHWs

**Figure 4:**
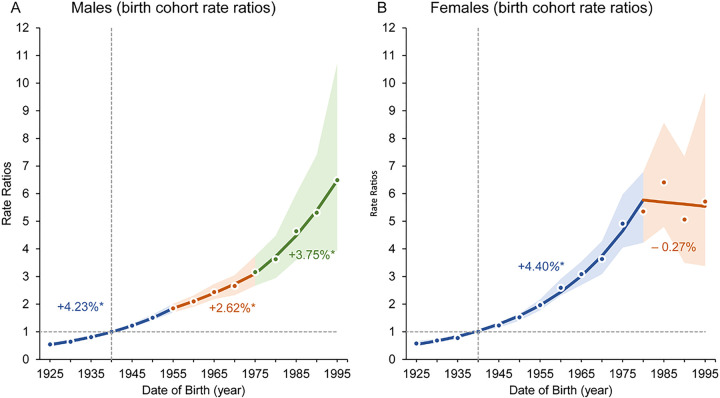
Incidence rate ratios of OTC by NHW birth cohort with Joinpoint regression trends. Incidence rate ratio by birth cohorts (A) among non-Hispanic White males and (B) among non-Hispanic White females. (*) indicates statistically Significant birth cohort percentage change in rate ratio for specific joinpoint segment. NHW: Non-Hispanic White; OTC: Oral tongue cancer.

Age-period-cohort analysis revealed increasing trends in OTC incidence among both NHW male and female birth cohorts between 1925 and 1995 (Fig. 4; Additional File 11: Figure S5 and Additional File 12: S6). The most recent birth cohort considered (individuals born between 1995 and 1999) had IRRs of 6.5 (95% CI, 3.9 to 10.7) for males and 5.7 (95% CI, 3.4 to 9.7) for females when compared to individuals of the same sex in the 1940 reference birth cohort. Both NHW males and females experienced IRR increases in successive birth cohorts from 1925 to 1955 (IRR_Male_ 4.2% [95% CI, 4.0–4.5%], IRR_Female_ 4.4% [95% CI, 4.1–4.7%]). Male IRR increases tempered slightly starting with the 1955 birth cohort, though Significant increases per successive 5-year birth cohort continued through the end of the observed period. Increases in successive female birth cohort IRRs maintained the same trajectory from 1925 until the 1980 birth cohort, at which point they stabilized across remaining birth cohorts. This mirrors our results from the earlier age-specific trends, which showed no Significant annual incidence rate increase among females under 30 years of age between 2001 and 2019.

### Case Projections for OTC Incidence among NHWs

**Figure 5:**
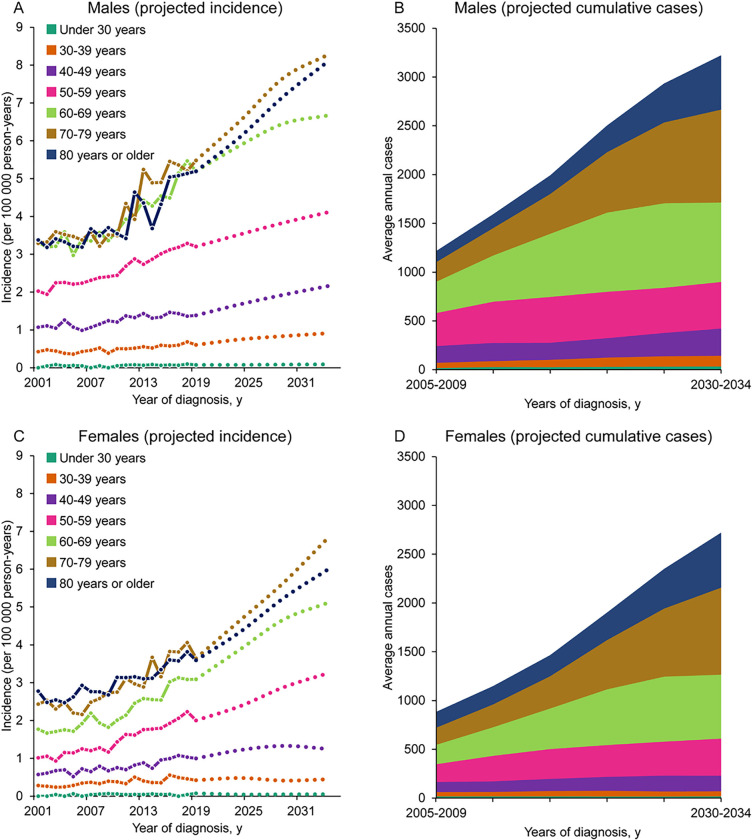
Age-specific OTC incidence trends and projections among NHW males and females. (A) Observed and projected age-specific incidence rates among males, (B) observed and projected cumulative case counts among males, (C) observed and projected age-specific incidence rates among females, and (D) observed and projected cumulative case counts among females. NHW: Non-Hispanic White; OTC: Oral tongue cancer.

The average annual number of new OTC cases among NHW persons is projected to be 5,997 per year by 2030–2034—a 73.4% increase over the average annual number of observed cases in 2015–2019 (Figs. 5B and 5D; Additional File 13: Table S7). Age-specific incidence rates (ASIR) are projected to rise by over 50% among males aged 40–49 years (1.4 to 2.2 per 100,000), females aged 50–59 years (2.0 to 3.3 per 100,000), and those aged 70–79 years and 80 years and older in both sexes (Males 70–79 years: 5.3 to 8.3 per 100,000; Males 80 + years: 5.0 to 8.1 per 100,000; Females 70–79 years: 3.7 to 6.8 per 100,000; Females 80 + years: 3.6 to 6.0 per 100,000) (Figs. 5A and 5C; Additional File 14: Table S8). This would result in the case burden skewing more heavily towards individuals aged 70 years and older (47% of total male cases in 2030–2034 versus 30% in 2015–2019, and 54% of total female cases in 2030–2034 versus 37% in 2015–2019). To ensure the shift in case burden was not simply the result of changing age demographics, we compared our projections to projected cases where 2015–2019 ASIRs remain fixed and are applied to future population estimates (Additional File 15: Table S9). Overall, females are projected to experience a larger increase in cases than males (86% increase versus 62% increase, respectively). While males were diagnosed with 35.9% more cases of OTC than females in 2015–2019, the above shifts combined with changing population age demographics project to shrink this discrepancy by almost half (to 18.4%) in 2030–2034.

## DISCUSSION

Our study is the first comprehensive analysis of nationwide OTC patterns using the 2001–2019 USCS Public Use Database, which covers nearly 100% of the U.S. population in all 50 states and the District of Columbia. Using this nationwide data, we are the first to show that the most substantial changes in OTC incidence rates have been concentrated in the Southeast region of U.S., particularly among Kentucky, Maryland, Tennessee and North Carolina. We are also the first to show a substantial shift in incidence rate increases and case burden from early onset OTC among NHW females to NHW females aged 50–59 years. We show through analysis of birth cohorts that rate increases have attenuated in NHW females born since 1980. Incidence rates among NHW males continued previously observed increases, with a Significant long-term trend of increase occurring among males aged 50 and older, but no substantial shift in overall case burden demographics. Altogether, this suggests a recent change in the yet to be identified risk factors underlying the previously documented rise in early onset cases among NHW females and calls for reevaluating the early-versus-typical onset paradigm that has dominated recent OTC literature. Using observed cases stratified by age and year of diagnosis, and accounting for the estimated distribution of the U.S. population over the next decade, we project continued and substantial increases in the total burden of new OTC incidence among both NHW males and females in future years. Should these trends continue over the next decade, new oral tongue cancer cases in the U.S. will increasingly be diagnosed among older individuals and the burden of disease will be shared more evenly between male and female patients than currently observed.

This study is consistent with and adds meaningful updates to prior research that found alarming increases in OTC incidence among younger NHW females in the U.S. In particular, Patel *et al*. showed rapid OTC incidence increases among 18–44 year old NHW females and stable rates among females > 44 years using data from 1975–2007.^3^ Tota *et al*. followed by finding rapid increases among NHW females < 50 years and the beginnings of increases in females ≥ 50 years using data from 1973–2012.^4^ In both studies, the younger age group was defined by individuals born after 1962. The current analysis adds an additional 7 years of cancer registry data (2001–2019), with some of the “younger females” identified by Patel *et al* and Tota *et al* now falling into the 50–59 years old age group. We report NHW females aged 50–59 as having experienced the largest increases in age-specific incidence—significantly outpacing increases among females less than aged 50. This, combined with the findings from our age-period-cohort analysis, suggests that a cohort effect may explain the previously observed increases in early onset OTC among females and argues for a shift away from the early versus typical onset dichotomy that has dominated OTC research over the last 20 years.

This study also shows similarities to patterns observed among oropharyngeal cancer in the U.S., both in terms of the geographic distribution of cases and the recently reported moderation of increasing OPSCC incidence rates among younger individuals.^15,23^ Currently, the factors driving OTC incidence increases remain unknown. Similarities with OPSCC incidence suggest there may be shared risk factors for developing these two subtypes of head and neck cancer. HPV infection as a common etiology is unlikely given prior studies demonstrating a lack of HPV detection in OTC tumors.^5,24,25^ Smoking prevalence cannot be ruled out without further analysis, but is also unlikely given the decreasing rates of other head and neck cancers as smoking rates have declined in the U.S.^1,2,13^ Researchers have hypothesized genetic factors, diet, immune deficiency, and environmental exposures as other potential causes.^12,13,26–29^ Our findings, which provide the first data on the geographic distribution of OTC incidence increases as well as the first evidence of a possible birth cohort effect, may help focus future research efforts towards identifying etiologic factors.

The results of this study have several meaningful implications for clinical practice. The profile of those at risk for OTC continues to evolve, with our findings indicating a need for heightened awareness and screening of older NHW persons and persons residing in the Southeast U.S. Surgical resection is the mainstay of treatment for OTC, which may be limited in older individuals, leading to a need for novel therapeutic approaches in older populations. Previous studies conducted under the paradigm of early-versus-typical onset OTC indicated that younger patients (and particularly those without traditional risk factors such as tobacco use) were at greater risk of recurrence and had higher rates of adverse pathological features.^26,30–32^ It is worth considering whether these characteristics will follow the same shift in age patterns as we report with incidence. It’s also worth noting that some studies have shown decreased relative and overall survival among OTC patients aged 50 or older.^11,33,34^ Lastly, although we have shown a leveling off in risk increases among younger NHW females, the overall rate of OTC among both NHW males and females remains much higher than historically observed and will continue to present at greater frequencies across patients of all ages.

Our study had several limitations. First, individual-level risk factors, such as HPV, smoking, obesity, and genetic conditions, are not routinely captured in cancer registries. Therefore, the impact of these and other risk factors on increasing incidence cannot be directly measured, leaving us to speculate on potential etiologies. Second, the possibility that HPV-related base of tongue cancers may have been misclassified as cancers of the oral tongue could lead to an overestimate of OTC incidence. To mitigate this risk, we excluded overlapping lesions of the tongue and malignancies not otherwise specified. The fact that observed increases in our study occurred in both NHW males and females while HPV-related base of tongue cancer has primarily been increasing in NHW males provides further evidence against the impact of possible misclassification. Third, limited case numbers among birth cohorts from 1990–2000 may result in unstable age-period-cohort estimates among younger individuals under 30 years old during the timeframe of this study. Fourth, the results of future case projections should be interpreted with caution, given limited observed case data among individuals under 20 years of age. Despite these limitations, the greatest strength of our study is the use of nationwide, high-quality, population-based registries that allowed us to perform analyses on the most comprehensive and contemporary OTC incidence data available in the U.S.

## CONCLUSIONS

This study shows an epidemiologic shift in recent patterns of OTC incidence in the U.S. Though rates of OTC are still broadly increasing across the population, the alarming rise of early onset cases among young NHW females appears to be giving way to rapid increases among females aged 50 years and older—indicating that previously observed increases seen among younger individuals could be attributable to a birth cohort effect. Given recent trends and the current age distribution of the U.S. population, the population of new OTC cases will likely become older and more evenly split between males and females over the next decade, which has important implications for future research as well as diagnosis and treatment.

## Figures and Tables

**Table 1:** Oral tongue cancer incidence (2001–2019) among non-Hispanic White persons: National Program of Cancer Registries and Surveillance, Epidemiology, and End Results Datasets

	Total		Trends	
Characteristics	Cases^a^, N (%)	Rate^b^ (95% CI)	AAPC (95% CI)	*P*
Overall	49072	1.03 (1.02, 1.04)	2.91% (2.18%, 3.65%)	< .001
Sex				
Male	28467 (58.0)	1.26 (1.25, 1.28)	2.58% (1.78%, 3.38%)	< .001
Female	20605 (42.0)	0.81 (0.80, 0.82)	3.61% (3.21%, 4.01%)	< .001
Age at Diagnosis				
Under 30 years	826 (1.7)	0.06 (0.05, 0.06)	2.44% (0.92%, 3.98%)	0.003
30–39 years	2041 (4.2)	0.43 (0.41, 0.45)	3.01% (2.31%, 3.71%)	< .001
40–49 years	5488 (11.2)	1.00 (0.97, 1.03)	2.44% (1.90%, 2.98%)	< .001
50–59 years	11561 (23.6)	2.06 (2.02, 2.09)	3.60% (3.26%, 3.93%)	< .001
60–69 years	13461 (27.4)	3.18 (3.12, 3.23)	3.31 % (2.47%, 4.15%)	< .001
70–79 years	9687 (19.7)	3.57 (3.49, 3.64)	2.93% (1.65%, 4.22%)	< .001
80 years or older	6008 (12.2)	3.40 (3.32, 3.49)	2.71% (2.21%, 3.21%)	< .001
Region^c^				
Southeast	12766 (26)	0.97 (0.96, 0.99)	4.77% (4.22%, 5.31 %)	< .001
Southwest	4268 (8.7)	0.94 (0.91, 0.96)	2.73% (2.13%, 3.33%)	< .001
Northeast	9492 (19.3)	1.00 (0.98, 1.03)	2.68% (2.05%, 3.33%)	< .001
Midwest	13029 (26.6)	1.07 (1.05, 1.09)	3.12% (2.11%, 4.15%)	< .001
Rocky Mountain	1671 (3.4)	0.94 (0.89, 0.98)	2.61% (1.52%, 3.72%)	< .001
Pacific	7144 (14.6)	1.22 (1.19, 1.25)	1.44% (0.83%, 2.04%)	< .001
Non-contiguous	187 (0.4)	1.14 (0.97, 1.32)	n/a	n/a
Stage at Diagnosis				
Local	31461 (64.1)	0.66 (0.65, 0.66)	1.93% (0.85%, 3.03%)	< .001
Regional	13283 (27.1)	0.28 (0.27, 0.28)	4.37% (3.22%, 5.52%)	< .001
Distant	2683 (5.5)	0.06 (0.05, 0.06)	2.77% (−2.71%, 8.56%)	0.98
Other/Unknown	1645 (3.4)	0.03 (0.03, 0.04)	2.15% (−0.07%, 4.41%)	0.057

a Cases include malignant tumors that matched selection criteria

b Rates were calculated as the number of cases per 100,000 person-years and age-adjusted to the 2000 US standard population;

c Southeast = Alabama, Arkansas, Delaware, District of Columbia, Florida, Georgia, Kentucky, Louisiana, Maryland, Mississippi, North Carolina, South Carolina, Tennessee, Virginia, West Virginia; Southwest = Arizona, New Mexico, Oklahoma, Texas; Northeast = Connecticut, Maine, Massachusetts, New Hampshire, New Jersey, New York, Pennsylvania, Rhode Island, Vermont; Midwest = Illinois, Indiana, Iowa, Kansas, Michigan, Minnesota, Missouri, Nebraska, North Dakota, Ohio, South Dakota, Wisconsin; Rocky Mountain = Colorado, Idaho, Montana, Nevada, Utah, Wyoming; Pacific = California, Oregon, Washington; Non-contiguous = Alaska, Hawaii. Cases diagnosed in Mississippi in 2001–2002 and in Nevada in 2018–2019 are not available. Cases diagnosed in Mississippi (Southeast region) in 2001–2002 and in Nevada (Rocky Mountain region) in 2018–2019 are not available

CI = Confidence Interval; AAPC = Average Annual Percentage Change

## Data Availability

The datasets supporting the conclusions of this article are included within the article (and its additional files).

## Abbreviations

APC: Annual percentage change
AAPC: Average annual percentage change
ASIR: Age-standardized incidence rate
CDC: Center for Disease Control and Prevention
CI: Confidence interval
HPV: Human papilloma virus
ICD-O-3: International Classification of Diseases for Oncology version-3
IRR: Incidence rate ratio
NCI: National Cancer Institute
NHB: Non-Hispanic Black
NHW: Non-Hispanic White
NPCR: National Program of Cancer Registries
OPSCC: Oropharyngeal squamous cell carcinoma
OTC: Oral Tongue Cancer
SEER: Surveillance, Epidemiology, and End Results
US: United States
USCS: United States Cancer Statistics
